# Subtype Transdifferentiation in Human Cancer: The Power of Tissue Plasticity in Tumor Progression

**DOI:** 10.3390/cells13040350

**Published:** 2024-02-17

**Authors:** Monica Fedele, Laura Cerchia, Sabrina Battista

**Affiliations:** Institute of Experimental Endocrinology and Oncology “G. Salvatore” (IEOS), National Research Council—CNR, 80131 Naples, Italy; cerchia@unina.it (L.C.); sabattis@unina.it (S.B.)

**Keywords:** luminal to basal-like transition, neuroendocrine differentiation, proneural to mesenchymal transition, tissue transdifferentiation, breast cancer, prostate cancer, glioblastoma, pancreatic adenocarcinoma

## Abstract

The classification of tumors into subtypes, characterized by phenotypes determined by specific differentiation pathways, aids diagnosis and directs therapy towards targeted approaches. However, with the advent and explosion of next-generation sequencing, cancer phenotypes are turning out to be far more heterogenous than initially thought, and the classification is continually being updated to include more subtypes. Tumors are indeed highly dynamic, and they can evolve and undergo various changes in their characteristics during disease progression. The picture becomes even more complex when the tumor responds to a therapy. In all these cases, cancer cells acquire the ability to transdifferentiate, changing subtype, and adapt to changing microenvironments. These modifications affect the tumor’s growth rate, invasiveness, response to treatment, and overall clinical behavior. Studying tumor subtype transitions is crucial for understanding tumor evolution, predicting disease outcomes, and developing personalized treatment strategies. We discuss this emerging hallmark of cancer and the molecular mechanisms involved at the crossroads between tumor cells and their microenvironment, focusing on four different human cancers in which tissue plasticity causes a subtype switch: breast cancer, prostate cancer, glioblastoma, and pancreatic adenocarcinoma.

## 1. Introduction

During development, determination and organization of cells in tissues is accompanied by their terminal differentiation, which locks cells into a specific phenotype, corresponding, in most cases, to a proliferative arrest [1]. One of the emerging hallmarks of cancer is the unlocking of phenotypic plasticity, which allows cancer cells to escape the terminally differentiated state and change their phenotype. Thus, a cancer cell initially committed to the differentiation pathway of the normal cell from which it originated, can, during tumor progression, switch to an entirely different developmental program, acquiring tissue-specific traits that were not preordained by the normal cell from which it originated [2]. In this tumor plasticity, the tumor microenvironment (TME), its crosstalk with tumor cells and the mechanisms of epigenetic reprogramming are involved [3]. Hypoxia and nutrient deprivation constitute the main pressure on epigenome change to achieve rapid adaptation to the hostile situation [4]. The passage from a differentiated phenotype to a different one can take place in two alternative ways: by returning to an undifferentiated state to then re-differentiate into a different subtype, or by direct transdifferentiation, in which cells completely change their developmental program, thereby acquiring tissue-specific traits that were not predestined to their normal cells of origin [2]. Here, by analyzing the most recent evidence, we will enter the specific molecular details examining four examples of subtype transition in human cancer: the luminal to basal-like transition in breast cancer (LBT), the neuroendocrine transdifferentiation (NET) of prostate cancer, the proneural-to-mesenchymal transition (PMT) in glioblastoma, and the acinar to ductal transition (ADT) in pancreatic carcinomas.

## 2. Luminal to Basal-Like Transition (LBT) in Breast Cancer

Breast cancer (BC) is a complex disease with significant heterogeneity, and subtyping is an important tool used to classify breast tumors into distinct molecular subtypes [5]. The two major subtypes are the luminal and the basal-like. The luminal subtypes are characterized by the expression of hormone receptors, such as estrogen receptor (ER) and/or progesterone receptor (PR). They account for most BC cases, and tend to have a more favorable prognosis compared to other subtypes. There are two main luminal subtypes: luminal A and luminal B. Luminal A tumors typically have a low proliferation rate and are associated with better outcomes. They have a high expression of hormone receptors (ER/PR) and a low expression of other markers associated with aggressiveness. Luminal A tumors are often sensitive to hormone-based therapies, such as endocrine therapy (e.g., selective estrogen receptor modulators or aromatase inhibitors). Luminal B tumors have a higher proliferation rate and tend to be more aggressive than Luminal A tumors. They often have lower expression levels of hormone receptors, and may also express other markers associated with increased proliferation or aggressiveness, such as HER2 (human epidermal growth factor receptor 2) or Ki-67 (a marker of cell proliferation). Luminal B tumors may require more aggressive treatment approaches, including chemotherapy in addition to endocrine therapy [5]. The basal-like subtype is often confused with triple-negative breast cancer (TNBC) because both lack the expression of hormone receptors (ER/PR) and HER2. TNBCs and basal-like tumors are generally associated with a poorer prognosis compared to luminal subtypes due to their aggressive nature and limited treatment options [6]. Basal-like tumors have a defined genetic profile characterized by high expression of cytokeratin 5, 6, 14, and 17, which are typically found in the basal cells of the mammary gland. Chemotherapy is the main treatment option for basal-like BC [7].

**Figure 1:**
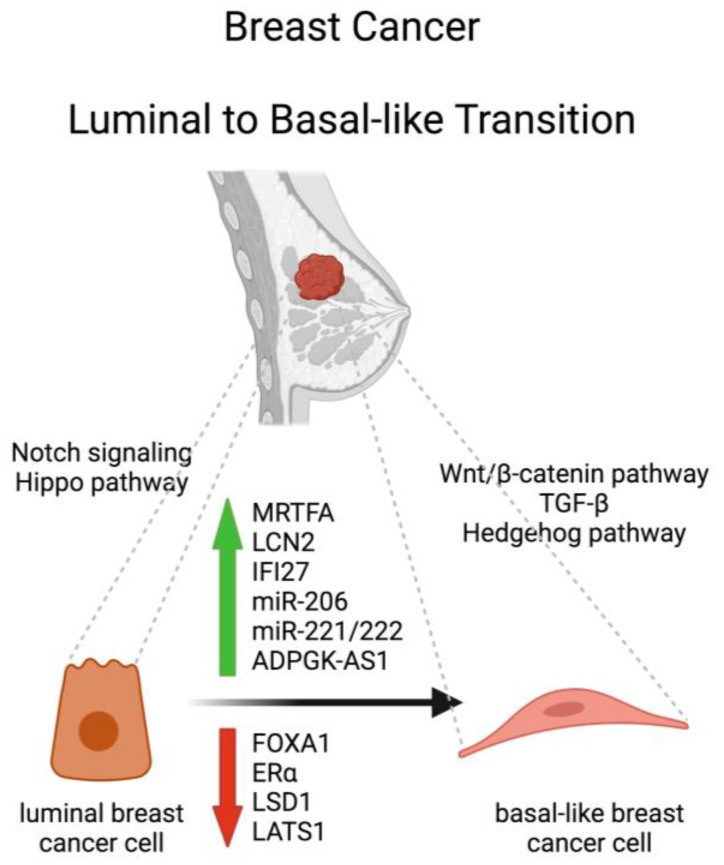
Schematic representation of molecules and signaling pathways involved in the luminal to-basal-like transition of breast cancer. FOXA1, Forkhead box protein A1; MRTFA, Myocardin-related transcription factor A; ERα, estrogen receptor alpha; LSD1, lysine-specific demethylase 1; LCN2, lipocalin 2; IFI27, interferon alpha-inducible protein 27; LATS1, Large Tumor Suppressor 1. Created with BioRender.

BC cells contain stem-, luminal, or basal-like phenotypes, which can interconvert [8]. The LBT in BC refers to a molecular and phenotypic change in tumor cells that undergo a transition from a luminal to a basal-like subtype. This transition is associated with a more aggressive phenotype, increased invasiveness, and poorer prognosis. Several molecular mechanisms are involved in driving this transition, including alterations in gene expression, signaling pathways, and crosstalk between the tumor cells and their microenvironment (Figure 1).

### 2.1. Transcriptional Regulation

Transcriptional regulation plays a crucial role in the LBT in BC. In fact, this transition involves the reprogramming of gene expression patterns, leading to the suppression of luminal lineage-specific genes and the activation of basal-like genes. Several transcription factors are involved in orchestrating these changes, and one key player in the LBT is the Forkhead box protein A1 (FOXA1), which plays a critical role in the development and differentiation of the mammary gland. It is predominantly expressed in luminal epithelial cells of the breast, and is involved in maintaining their luminal identity [9]. In luminal BC cells, FOXA1 binds to the enhancer regions of luminal lineage-specific genes, such as those encoding ER and GATA3, and facilitates the recruitment of other transcription factors and co-activators to these enhancer regions, promoting the expression of luminal-specific genes. However, during the luminal-to-basal transition, the activity of FOXA1 can be modulated, leading to its reduced binding to luminal gene enhancers. This results in the downregulation of luminal-specific genes and the loss of luminal identity. Along with the suppression of luminal genes, the luminal-to-basal transition involves the activation of basal-like genes. FOXA1 has been shown to participate in this process by binding to enhancer regions associated with basal-specific genes. Through its interaction with other transcription factors, such as GATA3 and AP-1, FOXA1 facilitates the recruitment of basal transcriptional machinery and activates the expression of genes associated with the basal-like phenotype. This includes genes involved in cell adhesion, migration, and epithelial-mesenchymal transition (EMT). Importantly, the balance between FOXA1 and other transcription factors determines the luminal or basal-like phenotype of breast cancer cells. Alterations in the expression or function of FOXA1, as well as changes in the activity of other transcription factors, can shift this balance and contribute to the LBT [9,10]. The Myocardin-related transcription factor A (MRTFA), alternatively known as Megakaryoblastic Leukemia 1 (MKL1), also plays a major role in the LBT of ERα positive BC cells, since its activation and nuclear accumulation mediate their endocrine resistance by initiating a partial transition from luminal to basal-like phenotype [11,12].

### 2.2. Epigenetic Modifications, Chromatin Remodeling, and Non-Coding RNAs

Epigenetic alterations, including DNA methylation and histone modifications, contribute to the LBT. Changes in DNA methylation patterns can silence luminal-specific genes, while activating basal-like genes. Histone modifications, such as histone acetylation and methylation, can also modulate gene expression patterns associated with the transition. ERα can directly silence basal markers involved in EMT, such as lipocalin 2 (LCN2) and interferon alpha-inducible protein 27 (IFI27) via DNA methylation. Moreover, by recruiting corepressors (CoRs) and HDACs that in turn recruit enhancer of zeste homolog 2 (EZH2) to modify histones with repressive H3K27me3 marks, ERα may direct DNA methylation-mediated silencing of a subpopulation of basal, stem, and EMT genes that potentially enforce luminal differentiation of BC cells [13]. Histone methylation is also involved in suppression of ERα during LBT. In fact, the lysine-specific demethylase 1 (LSD1) is enriched at the promoter region of the ERα gene and its reduction causes enhanced histone H3K9 methylation, which subsequently suppresses the transcription of the ERα gene. The reduced ERα expression leads to decreased epithelial characteristics of luminal BC cells [14].

The HMGA1 and HMGA2 proteins, architectural transcription factors that regulate the 3D chromatin structure facilitating the assembly of multiprotein complexes, promote BC LBT by activating stemness and key migration-associated genes, linked to the Wnt/beta-catenin, Notch and Pin1/mutant p53 signaling pathways [15,16].

In a recent study, the Large Tumor Suppressor 1 (LATS1), whose expression is often downregulated in human BC, has been reported to maintain luminal BC cell identity by reducing the chromatin accessibility of genes that are characteristic of a basal-like state. This is achieved via interaction of LATS1 with the Nuclear Core Repressive complex 1 (NCOR1) and recruitment of HDAC1, driving histone H3K27 deacetylation near NCOR1-repressed basal-like genes. Consequently, decreased expression of LATS1 elevates the expression of such genes and facilitates slippage towards a more basal-like phenotypic identity [17].

Non-coding RNAs, including microRNAs (miRNAs) and long non-coding RNAs (lncRNAs), have emerged as important regulators of the LMT [18,19]. Certain miRNAs, including miR-206 and miR-221/222, target luminal-specific genes, such as ERα, GATA3 and FOXA1, and inhibit their expression, thereby promoting the transition [18]. Conversely, some lncRNAs act as competing endogenous RNAs (ceRNAs) to sponge miRNAs and de-repress basal-like genes, such as vimentin and EMT transcription factors [20].

### 2.3. Signaling Pathways and Microenvironmental Factors

Various signaling pathways play a role in the LBT. The Notch signaling pathway, for example, has been implicated in promoting luminal differentiation. Inactivation of Notch signaling can lead to LBT [21]. Conversely, activation of pathways such as Wnt/β-catenin and TGF-β can promote the acquisition of basal-like characteristics. These pathways activate transcription factors, such as FOXC2, that drive the expression of basal markers [22,23]. Other signaling pathways include the Hippo and Hedgehog pathways, which counteract or sustain the basal-like state, respectively [24]. These pathways are activated by interactions with the TME [24,25]. The crosstalk between tumor cells and the TME, consisting of stromal cells, extracellular matrix components, and soluble factors, plays a crucial role in LBT. For instance, the presence of cancer-associated fibroblasts and tumor-associated macrophages (TAMs) can promote the transition by secreting factors and chemokines, like TGF-β and IL-8, which stimulate EMT and induce the acquisition of basal-like features [26,27,28].

## 3. Neuro-Endocrine Transdifferentiation (NET) of Prostate Cancer

**Figure 2:**
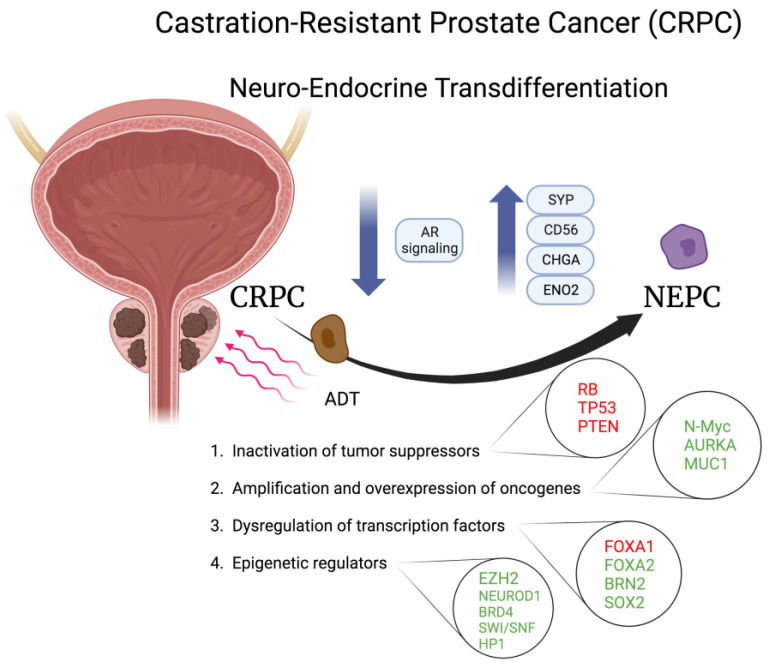
Schematic representation of molecules involved in the neuroendocrine transdifferentiation of prostate cancer. Molecules in red are inactivated or downregulated; molecules in green are amplified, activated, or overexpressed. CRPC, castration-resistant prostate cancer; NEPC, neuroendocrine prostate cancer; AR, androgen receptor; SYP, synaptoohysin; CHGA, chromogranin A; ENO2, enolase 2; AURKA, aurora A kinase; MUC1, mucin 1; FOXA1/2, Forkhead box protein A1/2; ADT, androgen deprivation therapy; BRN2. Created with BioRender.

Prostate cancer is the second leading cause of cancer-associated deaths among men in the United States [29] and ranks first among incident cancers of men in Italy [30]. Androgen deprivation therapy is the standard of care for advanced prostate cancer. However, the disease frequently progresses to a lethal metastatic form, defined as castration-resistant prostate cancer (CRPC), that in 20–25% of cases, after multiple rounds of ADT, transdifferentiates to neuroendocrine prostate cancer (NEPC). This is a lethal subtype of prostate cancer characterized by the expression of neuroendocrine (NE) markers such as chromogranin A (CHGA), synaptophysin (SYP), enolase 2 (ENO2) and CD56, together with the loss of androgen receptor (AR) signaling, thereby resulting in resistance to AR-targeted therapy, tumor aggressiveness and poor clinical outcome [29] (Figure 2). Some studies propose that the normal NE cells could be selected for survival during ADT and ultimately expand [31,32]. Other studies suggest that NEPC may arise from luminal cells, basal cells, or basal cells that lose their basal features and gain luminal features [33,34]. In the latter studies, one hypothesis is that NEPC may emerge from luminal-like tumor cells termed precursor cells in focal NEPC mixed with CRPC-adeno population, and ultimately progresses to the pure NEPC. Another hypothesis is that the NE phenotype is acquired when cells develop resistant mechanisms against AR-targeted therapies [29,35]. De novo NET has also been recently reported in primary untreated prostate cancer in association with advanced clinic-pathologic features and aggressive outcome [36]. Indeed, NEPC generally comprises a phenotype with morphologic and immunophenotypic transition from conventional adenocarcinoma towards high-grade neuroendocrine/small cell carcinoma and is associated with distinct genetic alterations and epigenetic programs, including inactivation of tumor suppressors, amplification and overexpression of oncogenes, dysregulation of transcriptional factors and epigenetic regulators (Figure 2).

### 3.1. Inactivation of Tumor Suppressors

Genomic analysis revealed that loss of RB, TP53 and PTEN are commonly associated with NEPC, suggesting they could be crucial for the NET from CRPC to NEPC. As a matter of fact, 70–90% and 60% of NEPC patients show loss of RB1 protein and PTEN, respectively. Moreover, mutation or deletion of TP53 is observed in 66.7% of NEPC patients compared with 31% of CRPC patients, and concurrent loss of RB1 and TP53 is observed in more than 50% of NEPC patients in comparison to ~14% of CPRC patients, suggesting a potential synergistic function in driving NEPC development [37]. Genetic mouse models confirmed that Rb1 and Trp53 cooperate to suppress prostate cancer lineage plasticity, metastasis, and antiandrogen resistance, since their loss induces NE phenotype through induction of SOX2 expression in prostate cancer initiated by the inactivation of PTEN [38,39].

### 3.2. Amplification and Overexpression of Oncogenes

Molecular characterization of NEPC identified the combined overexpression of N-Myc and Aurora A kinase (AURKA) as determinants of the NET. Indeed, N-Myc enhances AURKA stability, and upregulation of AURKA in LNCaP (AR-positive androgen-dependent cells) significantly upregulates NE marker NSE and EZH2, while decreasing AR expression, when compared to control cells [40]. Consistently, N-Myc amplification and overexpression is sufficient to drive NEPC initiated from human prostate epithelial cells [32]. Mechanistically, N-Myc binds to AR enhancers and forms an interaction with the AR that is dependent on its binding to EZH2, whose activity is enhanced by N-Myc. In this way, N-Myc cooperates with EZH2 to drive the NE phenotype in prostate cancer [41]. More recently, the cell surface receptor neurokinin-1 (NK1R) has been shown to be upstream of the AURKA/N-Myc signaling in driving NET in prostate cancer [42].

Another oncogene driving NET of prostate cancer is Mucin 1 (MUC1), whose amplification has been observed in ~30% of NEPC patients in comparison to 6% of CRPC patients [43]. Yasumizu et al. demonstrated that the upregulation of the oncogenic C-terminal subunit of MUC1, namely MUC1-C, in AR-dependent prostate cancer cells drives NEPC progression by repressing AR signaling, suppressing the p53 pathway, and inducing N-Myc and EZH2 expression [43]. Subsequently, the same research group showed that MUC1-C can drive NEPC progression also by activating the BAF (mammalian SWI/SNF) complexes in prostate cancer stem cells [44].

### 3.3. Dysregulation of Transcriptional Factors

As for breast cancer, FOXA1 has a critical role in prostate cancer cell plasticity. It directly interacts with AR and is critical for AR recruitment to enhancer sites in prostate cancer [45]. Its downregulation in LNCaP cells induces NE differentiation, while its overexpression in NEPC cells decreases the expression of the NE marker ENO2. Mechanistically, it suppresses IL-8 expression, which results in the induction of the MAPK/ERK pathway and inhibition of NE differentiation [46]. Conversely, FOXA2 expression is high in NE cell lines, metastatic NE tumor models, and NEPC patients [29]. Upregulation of FOXA2 is associated with the progression towards a NE phenotype by a Siah2-dependent concerted activity with HIF1α [47,48].

Another driver of NE phenotype in prostate cancer is BRN2, a master regulator of neural differentiation. It drives the NE phenotype by binding to SOX2, leading to its upregulation, and is directly downregulated by AR [49]. It can also be secreted in extracellular vesicles together with its cognate BRN4, which cooperates with BRN2 to drive NE phenotype by upregulating SOX2 expression [50]. Consistent with a critical role of SOX2 in NET, it is markedly upregulated in NEPC patients when compared to CRPC patients, and overexpression of SOX2 in LNCaP/AR cells induces the upregulation of NE and basal cell markers [39,49].

Achaete-scute homologue 1 (ASCL1) is another transcription factor playing a critical role in NET. Indeed, it is highly upregulated in NEPC compared to CRPC and its overexpression, induced by SOX2, enhances CRPC progression to NEPC by inducing cAMP-responsive element binding protein 1 (CREB1) phosphorylation which, in turn, mediates resistance to ferroptosis [51]. Moreover, overexpression of ASCL1 in prostate adenocarcinoma cells induces FOXA1 binding to NE regulatory elements and drives NE phenotype through histone modifications at their enhancer sites [52]. It is specifically involved in driving tumor heterogeneity during the progression to NPTC [53].

### 3.4. Epigenetic Regulators and Chromatin Remodelling

The histone methyltransferase EZH2, the catalytic subunit of Polycomb repressive complex 2 (PRC2), inhibits the transcription of downstream target genes mainly by methylation of H3K27, and it has been shown to be significantly overexpressed in NEPC compared to other prostate cancer clinical samples [54]. Overexpression of RacGTP enzyme activating protein 1 (RACGAP1) by E2F1 was recently shown to contribute to neuroendocrine transdifferentiation of prostate cancer [55]. Mechanistically, RACGAP1 stabilizes EZH2 expression in the ubiquitin-proteasome pathway which, in turn, drives the transdifferentiation [55]. More generally, different chromatin modifiers, including NEUROD1, BRD4, SWI/SNF and HP1 family, play a role in lineage reprogramming of prostate adenocarcinomas to NEPC under the selective pressure of various AR-targeted therapies [56].

The already mentioned ASCL1 transcription factor plays a pivotal role in the early chromatin remodeling involved in driving NET of prostate cancer through the regulation of neuronal and stem cell programs. In more detail, ASCL1 activates transcription of UHRF1 that binds to AMPK to stabilize PRC2 complex and enhance H3K27-trimethylation. Loss of ASCL1 indeed inhibits EZH2 activity and chromatin remodeling. This switches neuroendocrine phenotype to luminal subtype [57].

## 4. Proneural-to-Mesenchymal Transition in Glioblastoma

Glioblastoma (GBM) (grade IV glioma) is the most common and aggressive form of brain tumor, with a survival of only 15 months from the diagnosis. Its high invasiveness and resistance to conventional therapies makes GBM a still incurable ailment, notwithstanding the strenuous search for a cure. GBM, far from being a uniform disease, takes place in at least three different tumor-intrinsic subtypes—Classical (CL), Proneural (PN) and Mesenchymal (MES)—each one defined by a characteristic molecular signature, phenotype, and prognosis [58,59], with PN and MES being the most severe and the MES having the worst prognosis. Even though subtypes may coexist within the same tumor, mirroring the cellular and spatial heterogeneity of GBM [60,61], chemoradiation may induce transition from one subtype to another, mainly from PN to MES, contributing to therapeutic resistance [62].

Whereas the PN subtype has been defined as a “hardwired identity”, the MES GBM is an adaptive and evolving state, often arisen from PN tumors [63] triggered by microenvironmental, pro-inflammatory and DNA damage signals. Innate immune cells divert PN subtypes to MES, conferring therapeutic resistance [64].

Even though the presence of different populations of various subtypes in GBMs undermines the clinical value of GBM classification, an extensive knowledge of the transcriptional signature of GBM may contribute to determine the pathogenetic molecules involved, potentially useful in diagnosis, prognosis, and targeted therapy [65].

Seminal studies have established that PN GBMs are characterized by IDH1 mutations, the glioma-CpG island methylator phenotype (G-CIMP+) [58,66], PDGFRA amplification and OLIG2 expression [58,67], whereas MES GBMs are G-CIMP− and share wild-type IDH1, loss of NF1 as well as the expression of CD44, signal transducer and activator of transcription 3 (STAT3) and CCAAT enhancer binding protein beta (CEBPβ) [58,66,68], which finally lead to increased proliferation, resistance to radiation and worse prognosis [69]. However, the following three groups of adult-diffuse gliomas are recognized in the 2021 WHO classification: (1) astrocytoma, IDH-mutant, graded 2–4; (2) oligodendroglioma, IDH-mutant and 1p/19q-codeleted; and (3) glioblastoma, IDH-wildtype. In addition, if an IDH-mutant diffuse astrocytoma exhibits CDKN2A/B homozygous deletion, it is designated as a CNS WHO grade 4 neoplasm [70]. Within each group, it is possible the subtyping in CL, PN and MES phenotype, with PN being most dominant in IDH-mutant tumors and MES more common among IDH-wildtype tumors [71].

### 4.1. Role of Stem Cells in PMT

GBM arises from a small sub-population of cancer cells with stem-like properties (glioma stem cells or GSCs) [72], derived, upon tumorigenic mutations, from normal neural stem cells (NSCs) in the subventricular zone (SVZ) [73]. GSCs and NSCs share common stemness and proliferation pathways, such as the Notch pathway, the Sonic Hedgehog pathway, SOX2, STAT3, Wnt, β-catenin, POU3F2, SALL2 [73,74], and CD44 [75].

Importantly, GSCs isolated from GBM were shown to be able to develop GBM in transplanted mice [76,77] and to be responsible of GBM progression, resistance to therapies and relapse [73]. Being the source of all the cancer cells within the tumor, GSC characteristics affect GBM molecular and phenotypic properties, including molecular markers and resistance features [75,78,79]. For this reason, GSCs are the object of intense research, aimed at characterizing their properties and molecular signatures. Seemingly, the subtype characteristics are determined by founder GSCs and by their successive genetic and epigenetic modifications.

Accordingly, mRNA profiles have identified PN and MES GSCs, overlapping the respective PN and MES GB profiles [79], with MES GSCs being characterized by higher malignancy and radio resistance and by an associated immunosuppressive microenvironment [59,80].

However, it was found that isolated GSCs differ in the transcriptome and epigenetic profiles when compared to the originating tumor, even though they form tumors that were histologically similar to the tumor of origin when xenotransplanted [75].

GSCs were shown to be able to acquire mesenchymal features, starting from a proneural phenotype through the activation of the TNF-α/NF-κB pathway and downstream transcription factors, as well as the enrichment of CD44. This phenomenon recapitulates the epithelial-mesenchymal transition process characterizing the transition of normal basal glia cells to basal intermediate progenitors (iBP), during brain development [81].

However, even though transcriptomic analyses have identified specific markers, mutations and epigenetic modifications following radiation therapy and associated with PMT of GSCs, it is not clear if the PN-to-MES transition is determined by MES GSCs already present in the heterogeneous tumor bulk, taking over on the PN GSCs due to their intrinsic resistance to therapy, or if triggered by treatment, pushing the cells to change profile [69].

Factors responsible of PMT span intracellular and microenvironmental cues, such as chemokines and their cognate membrane receptors, signaling kinases and cytoskeletal proteins [82], epigenetic and transcription factors, cell cycle controllers, immune cells, and possibly other factors.

Strikingly, even though different drivers converge in promoting PMT, each of them seems to activate a specific transcriptional signature, still partially overlapping with the transcriptional output induced by the others [64].

### 4.2. Epigenetic and Transcription Factors

Transcription factors determine cell phenotype by binding to cis-regulatory units of the DNA. They are also able to reprogram differentiated GBM cells into GSCs and to determine GBM subtypes [68,74,83]. Accordingly, PMT takes place upon epigenetic modifications and activation of transcription factors. Seminal studies have shown that the PN GBM subtype is characterized by a G-CIMP phenotype [66] distinct from a MES/G-CIMP^−^ signature. This PN/G-CIMP^+^ phenotype also characterize most GSCs isolated from predominantly MES/G-CIMP^−^ GBMs [75].

Seemingly, epigenetic modifications also regulate the expression of transcription factors that have been shown to be overexpressed in MES GBMs, supporting the PMT by binding to target promoters. Examples are STAT3, c/EBP-β [68], the transcriptional coactivator with PDZ-binding motif (TAZ) [84], the Runt-related transcription factor 1 (RUNX1) [84], and the Fos-like antigen 1 (FOSL1), composing the AP1 transcription factor complexes [85]. All these transcription factors are upregulated in MES GSCs compared with PN GSCs and neural progenitors and, by spurring the invasive process, sustain MES GBM and its poor prognosis. Besides fostering the MES subtype, they allow ionizing radiation (IR)-induced PMT and radioresistance of GSCs, whereas their silencing in MES GSCs inhibits tumorigenic ability and MES characteristics, including expression of mesenchymal markers and invasive phenotype.

### 4.3. Post-Transcriptional Mechanisms (Non-Coding RNAs and Proteostatic Mechanisms)

Recently, RNA-mediated mechanisms have been evaluated, and splicing profiles of GSC lines have shown significant differences between the PN and MES subtypes, involving genes implicated in cell cycle regulation, DNA repair, cilium assembly, and RNA splicing. In addition, some lncRNAs were shown to be differentially expressed in the two subtypes, with some of them having prognostic value [86].

Several lncRNAs are involved in PMT, conceivably upregulated in chemo- and radio-resistant GSCs. This is the case of the lncRNA PDIA3P1, which was found upregulated in MES GBMs, fostering PMT and conferring resistance to temozolomide (TMZ). Mechanistically, it was found that p38α-MAPK signaling pathway mediates the TMZ-induced upregulation of PDIA3P1 which, in turn, stabilizes c/EBPβ by inhibiting its MDM2-mediated ubiquitination [87]. Similarly, the lncRNA MIR222HG, highly expressed in MES GBM tissues, binds to and activates the YWHAE/HDAC5 complex, favoring H4 deacetylation, PMT and radio resistance [88].

Alteration of proteostasis through the damage of the ubiquitin-mediated proteasome degradation system also plays a role in PMT. It has been shown that the deubiquitinating enzyme USP10 is overexpressed in MES GBs where it stabilizes RUNX1 and promotes PMT. Accordingly, USP10 was found to have a prognostic value in GBM patients [89].

In addition, PMT has been shown to be induced by UBC9-dependent SUMOylation of CYLD, leading to the polyubiquitination and activation of NF-κB [90].

### 4.4. Metabolic Alterations in PMT

In recent years, the tight bi-directional interconnection between cancer and metabolism has been evidenced, showing that whereas oncogenesis transformation inevitably affects cancer cell metabolism, metabolism per se dramatically impacts carcinogenesis [91]. Strikingly, metabolism and metabolic enzymes may also affect the subtype switching. This is the case of ALDH1A3, belonging to the aldehyde dehydrogenase (ALDH) family genes, and the glycolytic pathway, which were found upregulated in MES GSCs [79]. More recently, Schmitt and colleagues have highlighted a switch in the metabolic transcriptome associated with PMT and involving genes of cholesterol biosynthesis and the SREBP1/2 pathway. Accordingly, not only oxidized Low Density Lipoprotein (oxLDL), but also Nitric Oxide (NO), was able to induce mesenchymal drift in GSCs [64].

Similarly, pyrroline-5-carboxylate reductase 2 (PYCR2), which is involved in proline synthesis together with the AlkB homolog 5 RNA demethylase (ALKBH5), promotes PMT, cell proliferation, migration, and invasion in GBM [92].

### 4.5. GBM Microenvironment: Immune Cells, Chemokines, and Extracellular Vesicles

As for other types of cancer, the GBM tumor bulk is constituted not only by tumor cells, but also by diverse infiltrating and local host cells, secreted chemokines, and an extracellular matrix, which are collectively designated as the microenvironment [93]. The GBM microenvironment is continuously modified by the cancer cells, which determine the composition in terms of cells (stromal, immune and blood vessels), and extracellular matrix. In turn, tumor microenvironment affects tumor progression and characteristics, including subtype transition.

The importance of GBM microenvironment in GSC PMT was highlighted by Bath and colleagues, showing that GSCs isolated from MES GBMs express PN signature (even in cells with a “MES” genotype), but can undergo MES differentiation upon TNF-α-mediated NF-κB activation [75].

Oncostatin M (OSM), which plays a key role in tumorigenesis and poor prognosis, has been shown to promote PMT by affecting the Jak-STAT and NF-κB pathways and modifying the tumor microenvironment [94].

The C-X-C Motif Chemokine Receptor 4 (CXCR4), the receptor for SDF-1, which promotes EMT in several cancers, has been shown to increase the expression of MES genes in glioma cell lines and its higher expression correlates with significantly reduced survival in GBM [95].

Moreover, whereas NF1 hypermutation is related to CD8^+^ T cell enrichment, loss of NF1 in MES GBs increases the tumor-associated macrophages/microglia infiltration; accordingly, M2 macrophages detection is associated with rapid relapse after radiation therapy [59].

**Figure 3:**
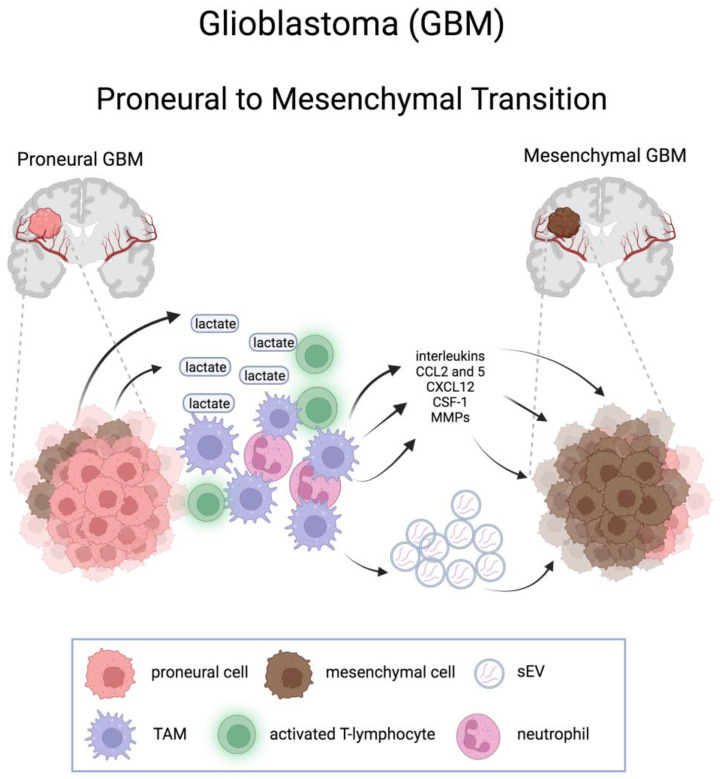
Schematic representation of the effects of tumor microenvironment on the proneural-to-mesenchymal transition in glioblastoma. CCL2 and 5, C-C motif chemokine ligand 2 and 5; CXCL12, CXC motif chemokine ligand 12; CSF-1, macrophage colony-stimulating factor 1; sEV, small extracellular vesicle; TAM, tumor associated macrophage. Created with BioRender.

Conversely, a causal connection exists between innate immune cell infiltration and mesenchymal transdifferentiation, leading to the acquisition of selective resistance to therapies [64]. Depletion of monocytes, as well as their conversion to macrophages, has been shown to promote PMT [96,97], through the stimulation of intratumoral neutrophils flux and neutrophil-derived TNF-α which, in turn, increases hypoxia in PDGFB-driven GBMs and fosters PMT [96]. A local inflammatory reaction consisting of various immune cells, including TAMs, is also a consequence of an acidic microenvironment resulting from the increased production and release into the extracellular space of lactate from MES GBM cells due to the increase in glycolytic activity [79]. These cells secrete cytokines and growth factors that promote PMT [98]. TAMs would exert their action on PMT at least in part also by secreting small extracellular vesicles (sEV), which transfer key microRNA molecules to GSCs [97] (Figure 3).

EVs are also produced by tumor apoptotic cells (apoptotic extracellular vesicles or apoEVs): they have been shown to affect both proliferation and phenotype of surviving cancer cells and to induce PMT. Mechanistically, splicing factors and components of the spliceosome, overexpressed in apoEV, become internalized in adjacent cancer cells, favoring MES-like type of splicing in PN cells [99].

### 4.6. Reversal of PMT

As mentioned before, GSCs have been found to revert to a PN phenotype when isolated from their MES originating tumors [75]. Accordingly, in vitro and in vivo synthetic genetic tracing studies demonstrated that the interconversion between PN and MES states is bidirectional [64]. As for the PMT, genetic mechanisms have been identified also for its reversion. An example is provided by ASCL1, a member of the basic helix–loop–helix (BHLH) family of transcription factors that, by activating a PN signature and downregulating the expression of N-Myc downstream-regulated gene 1 (NDRG1), spurs a neuronal-like differentiation of GSCs [100], suppresses self-renewal and decreases tumorigenesis [101].

Given the better prognosis of PN tumors compared to MES, it is intuitive that targeted therapies able to revert the PMT might be effective GBM treatments. In line with this idea, using orthotopic GBM tumors in mice, it has been shown that engineered exosomes from human bone marrow mesenchymal stem cells (MSCs) encapsulating siRNAs targeting Myc (iExo-Myc) inhibit proliferation and angiogenesis, revert PMT, suppresses tumor growth, and extends survival [102]. Similarly, STAT3 inhibitors suppress the OSM-mediated biological effects, including PMT, in GBM cells [94].

As we have previously seen, the upregulation of the lncRNA PDIA3P1 promotes PMT and is regulated by the p38α-MAPK signaling pathway. The small p38α-targeting drug Nefllamapimod (NEF), hampers TMZ-responsive PDIA3P1 overexpression and synergize with TMZ both in vitro and in vivo [87].

## 5. Acinar-to-Ductal Transdifferentiation in Pancreatic Carcinomas

**Figure 4:**
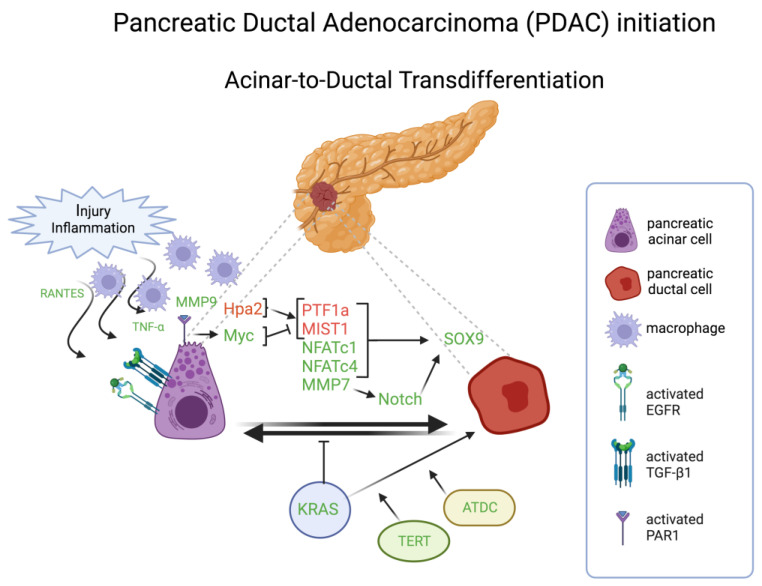
Schematic representation of the major molecules involved in the acinar-to-ductal transdifferentiation in pancreatic ductal adenocarcinoma initiation. Molecules in red are inactivated or downregulated; molecules in green are amplified, activated, or overexpressed. TNF-α, transforming growth factor-α; MMP, metalloprotease; Hpa2, heparanase; PTF1a, pancreas associated transcription factor 1a; MIST1, muscle, intestine, and stomach expression 1; NFAT, nuclear factor of activated T cells; ATDC, ataxia-telangiectasia group D-complementing. Created with BioRender.

One illuminating case for transdifferentiation as a discrete event in tumorigenesis involves pancreatic ductal adenocarcinoma (PDAC), wherein one of the implicated cells of origin, the pancreatic acinar cell, can become transdifferentiated into a ductal cell phenotype during the initiation of neoplastic development (Figure 4). PDAC has a low 5-year survival rate of below 11% [103], and is projected to be the second leading cause of cancer-related mortality in the United States and Europe by 2030 [104]. One of early events in PDAC tumorigenesis is the acinar-to-ductal transdifferentiation, often referred to as acinar-to-ductal metaplasia (ADM), followed by pancreatic intraepithelial neoplasia (PanIN), leading to invasive carcinoma. ADM is generally a reversible healing process triggered by pancreatic injury or inflammation, in which acinar cells transform into ductal progenitor-like cells, undergo proliferation, and contribute to the restoration of lost or damaged tissue. As the healing process unfolds, ductal progenitor cells revert to acinar cells, restoring normal acinar function. Nevertheless, the presence of oncogenic KRAS mutations hinders this redifferentiation process, resulting in persistent metaplasia. This persistence allows the progression to neoplastic lesions and ultimately PDAC [105,106,107].

### 5.1. Trancriptional Factors

Two transcriptional factors, pancreas associated transcription factor 1a (PTF1a) and muscle, intestine, and stomach expression 1 (MIST1), govern, through their expression in the context of self-sustaining, “feed-forward” regulatory loops, the specification and maintenance of differentiated pancreatic acinar cell state [108]. Both transcriptional factors are often downregulated during neoplastic development and malignant progression of human and murine PDAC. Functional genetic studies in mice and cultured human PDAC cells demonstrated that experimentally forced expression of PTF1a impairs KRAS-induced transdifferentiation and proliferation, and can also force the re-differentiation of already neoplastic cells into a quiescent acinar cell phenotype [109]. Conversely, suppression of PTF1a expression results in acinar-to-ductal metaplasia, i.e., transdifferentiation, and thereby sensitizes duct-like cells to oncogenic transformation by KRAS, accelerating subsequent development of invasive PDAC [110]. Similarly, using an in vitro three-dimensional (3D) culture system to model ADM outside the animal, forced expression of MIST1 in KRAS-expressing pancreas also blocks transdifferentiation and impairs the initiation of pancreatic tumorigenesis otherwise facilitated by the formation of duct-like precancerous lesions (PanIN), whereas genetic deletion of MIST1 enhances their formation and initiation of KRAS-driven neoplastic progression [111]. Loss of either PTF1 or MIST1 expression during tumorigenesis is associated with elevated expression of another developmental regulatory transcription factor, SOX9, which is normally operative in ductal cell specification [110,111]. Forced upregulation of SOX9, obviating the need to downregulate PTF1a and MIST1, has also been shown to stimulate transdifferentiation of acinar cells into a ductal cell phenotype susceptible to KRAS-induced neoplasia [112], implicating SOX9 as a key functional effector of their downregulation in the genesis of human PDAC (Figure 4). Thus, three transcription factors that regulate pancreatic differentiation can be variously altered to induce a transdifferentiated state that facilitates, in the context of KRAS mutational activation, oncogenic transformation and the initiation of tumorigenesis and malignant progression. Additional members of the SOX family of chromatin-associated regulatory factors are on the one hand broadly associated with both cell fate specification and lineage switching in development [113], and on the other hand with multiple tumor-associated phenotypes [114].

Other transcriptional factors playing a crucial role in pancreatic cell plasticity are members of the inflammatory-dependent nuclear factor of activated T cells (NFATc) family. Among them, NFATc1 is rapidly and transiently induced in early adaptation to acinar cell injury in human samples and in mice, where it promoted ADM by linking EGFR signaling to induction of SOX9 [115,116]. Another member, NFATc4, which is highly induced and localizes in the nucleus in response to inflammation-induced EGFR signaling, also drives ADM and PDAC initiation through direct transcriptional induction of SOX9 [117].

### 5.2. Oncogenic Signaling and Tumor Suppressor Genes

Apart from the already described role of the KRAS oncogene, under in vitro two-dimensional (2D) culture conditions, human and mouse acinar cells can transform into duct-like cells and can undergo transdifferentiation to metaplastic ductal epithelial cells in response to TGF-α and EGFR signaling [118]. Molecular dissection of this process in vitro has shown that primary acinar cells, in response to EGF receptor ligands, can transdifferentiate into duct-like epithelia, passing through a nestin-positive intermediate, in a Notch pathway-dependent manner which is dependent on matrix metalloproteinase 7 (MMP-7) [119,120]. Also, in in vitro 3D cultures, transforming growth factor β1 (TGF-β1) promotes ADM of human acinar cells [121]. Transgenic mice overexpressing transforming growth factor-α (TGF-α) in the pancreas show a transdifferentiation of acinar cells toward duct-like cells [122]. KRAS-induced ADM is accelerated in acinar cells expressing high levels of TERT, which causes clonal expansion of acinar cells that serve as a reservoir for the accumulation of additional genetic and epigenetic changes necessary for the transdifferentiation [123]. Moreover, the ataxia-telangiectasia group D-complementing (ATDC) gene is required for KRAS-driven ADM and its progression to PanIN through activation of β-catenin signaling and subsequent SOX9 up-regulation [124].

A player essential in pancreatic ADM is the Heparanase 2 (Hpa2). It binds heparane sulfate (HS) but, unlike Hpa, cannot degrade HS, which is considered a protumorigenic function [125], but acts as a competitor of Hpa, thereby inhibiting its enzymatic activity [126]. Therefore, it has been suggested to be a tumor suppressor of PDAC. Consistently, the lack of Hpa2 in knockout mice was associated with a marked decrease in the expression of key pancreatic transcription factors such as PTF1, GATA6, and MIST1, resulting in ADM [127]. Another tumor suppressor pathway inactivated by oncogenic KRAS that could be responsible for pancreatic ADM is the SAG-SHOC2 axis. In a primary 3D culture. Tan and colleagues demonstrated that SAG deletion induces ADM due to the resulting SHOC2 accumulation which, in turn, induces MAPK and mTORK1 pathways [128].

### 5.3. Metabolic Pathways

Activating mutations in KRAS extensively reprogram cellular metabolism to support the continuous growth, proliferation, and survival of pancreatic tumors. The metabolic reprogramming required for the pancreatic ADM has been explored by Radik and colleagues through transcriptomic analysis on mouse acinar cells undergoing ADM [129]. Metabolic pathways are globally enhanced during the ADM. Among them, NRF2-target genes, including those coding for the two rate-limiting oxidative PPP enzymes, Glucose-6-phosphate dehydrogenase (G6PD) and 6-phosphogluconate dehydrogenase (PGD), are upregulated. However, G6PD deficiency in animal models leads to decreased oxidative PPP flux, causing increased reactive oxygen species (ROS) and acceleration of the transition [129].

The role of glycogen synthase kinase-3beta (GSK-3β) in ADM has been studied by Ding and colleagues, showing that GSK-3β promotes TGF-α-induced ADM in 3D cultured primary acinar cells, whereas deletion of GSK-3β attenuates caerulein-induced ADM formation and PanIN progression in Kras^G12D^ transgenic mice by suppressing oncogenic KRas-driven cell proliferation through increasing the activation of S6 kinase [130].

A key metabolite in governing ADM is cholesterol. Consistently, cholesterol supplementation on isolated primary wild-type acinar cells enhances ductal transdifferentiation, associated with generation of the second messenger cyclic adenosine monophosphate (cAMP) and the induction of downstream protein kinase A (PKA) [131].

### 5.4. Microenvironment Crosstalk

Macrophages are key players in ADM. Macrophage-secreted matrix metalloproteinase (MMP)-9 induces ADM by binding to protease-activated receptor 1 (PAR1) and activating Myc on acinar cells. Consistently, PAR1 deficiency, as well as Myc inhibition, limits ductal transdifferentiation in experimental in vivo and in vitro systems for ADM, and silencing PAR1 or inhibiting Myc in PDAC cells re-establishes acinar cell identities in these ductal cells. This suggests that also ductal cells are plastic and able to regenerate into acinar-like cells, even in the presence of oncogenic KRAS activation. PAR1-Myc axis inactivation leads to increased expression levels of PTF1A and MIST1, whereas expression levels of SOX9 and KRT19 decrease [132]. Moreover, macrophages infiltrating the pancreas drive ADM by secreting inflammatory cytokines RANTES and TNF-α, which induce ADM through activation of NF-κB and its target genes, including MMPs, involved in regulating survival, proliferation, and degradation of extracellular matrix [133] (Figure 4).

## 6. Clinical Perspectives

In the field of clinical oncology, the phenomenon of tumor subtype transdifferentiation has emerged as a pivotal and dynamic aspect influencing cancer progression and therapeutic approaches. Tumor cells, traditionally classified into distinct subtypes based on their histological and molecular features, exhibit a remarkable capacity to undergo transdifferentiation, wherein they acquire characteristics that mark different subtypes. This inherent plasticity challenges the conventional understanding of cancer heterogeneity and has significant implications for diagnosis, prognosis, and treatment strategies. In the latter case, understanding the mechanisms driving transdifferentiation is crucial for developing targeted therapies that can effectively address the evolving landscape of tumor subtypes, ultimately contributing to improved patient outcomes. In the clinical practice, there are currently no specific targeted therapies explicitly designed to block tumor subtype transdifferentiation, but several studies in recent years highlighted the importance of tumor subtype transitions in both diagnosis and therapy. Just to mention some of the most promising studies focused on the tumors here described, Wang et al. have used single cell RNA sequencing profiling to establish a transcriptomic map of NEPC transdifferentiation. They identified two NE programs with related but distinct gene expression patterns and TF networks. The dynamics of NE gene expression and NEPC’s relationship with ADPC provide instrumental knowledge in designing more informed diagnosis strategies in clinical practice [134]. Independently from many genetic predisposition factors found in NEPC, the sphingosine kinase 1/sphingosine-1-phosphate (SphK1/S1P) signaling, being pivotal for normal physiology of neurogenesis, lymphocyte trafficking and vascular development through roles in cell proliferation, survival, differentiation, motility and intracellular calcium regulation, plays an autocrinal role in NEPC onset from CRPC [135]. Mechanistically, S1P activates the MAPK pathway to increase the phosphorylation of RE-1 silencing transcriptional factor (REST), leading to its rapid turnover by proteasome degradation, which unleashes transcriptional repression of neuronal transcriptional factors expression. Consistently, a significant correlation between prostate cancer with Shpk1^high^/REST^low^ with the poor overall survival (OS) of patients has been observed [135]. Similarly, the expression of SphK1 is positively correlated with poor OS and progression-free survival (PFS) of breast cancer [136]. Importantly, Lee et al. showed that FDA-approved SphK1-specific inhibitors (FTY720 or SKI-II) can overcome Enzalutamide-resistant CRPC tumor growth, suggesting that the repurposing of these small molecules has an immediate translational applicability to improve the outcome of NEPC patients to prolong their OS [135].

As already mentioned, alteration of proteostasis also plays a role in subtype transition of GBMs, where the overexpression of the deubiquitinating enzyme USP10 induces PMT and correlates with a worst prognosis [89]. Conversely, reversal of PMT by inhibiting Myc, suppresses GBM orthotopic tumor growth and extend survival of mice [102]. Moreover, Lee et al. demonstrated that the inhibition of macrophage migration inhibitory factor (MIF) and D-dopachrome tautomerase (DDT) by 4-iodo-6-phenylpyrimidine (4-IPP), a dual inhibitor targeting MIF and DDT, downregulates stemness phenotype, intracellular signaling cascades, MES transdifferentiation, and induces apoptosis in PN GSCs. In vivo preliminary results using a subcutaneous xenograft model demonstrated a significant tumor-suppressing effect of 4-IPP when combined with radiation therapy. Collectively, the targeted inhibition of MIF and DDT has the potential to strengthen current clinical strategies of GBM by enhancing the anticancer effects of radiation therapy [137].

## 7. Conclusions

Tumor cells exhibit remarkable plasticity, enabling them to transdifferentiate and adapt to changing microenvironmental landscapes, with profound implications for disease progression, response to treatment, and clinical outcomes. This dynamicity, driven by phenotypic variations and influenced by microenvironmental factors, has emerged as a critical aspect of cancer biology. As a result, the traditional classification of tumors into discrete subtypes is increasingly insufficient to capture the complexity of cancer, especially in the era of next-generation sequencing. In particular, the integration of next generation analyses, such as single cell spatial RNA sequencing, spatial transcriptomic and pseudo-time analysis, has identified even more complex scenarios, with different cellular subtypes co-existing in the same sample and differentiation trajectory of tumor cells from one subtype to another [138]. The insights presented here, with a specific focus on breast cancer, prostate cancer, glioblastoma, and pancreatic adenocarcinoma, highlight the importance of studying tumor subtype transitions. Understanding the underlying molecular mechanisms driving these transitions is essential for advancing our knowledge of tumor evolution and developing personalized therapeutic approaches.

## Data Availability

Not applicable.
